# Supplementary material for: PIP5Kγ Mediates PI(4,5)P2/Merlin/LATS1 Signaling Activation and Interplays with Hsc70 in Hippo–YAP Pathway Regulation
Source: Int J Mol Sci. 2023 Sep 30;24(19):14786. doi: 10.3390/ijms241914786 (PMC10572892; doi:10.3390/ijms241914786)
Supplement: Supplementary file 1 [file ijms-24-14786-s001.zip › Supplementary materials (revised).pdf]

Supplementary Materials

PIP5Kγ Mediates PI(4,5)P2/Merlin/LATS1 Signaling Activation and Interplays with Hsc70 in Hippo–YAP Pathway Regulation

Duong Duy Thai Le [1], Truc Phan Hoang Le [1] and Sang Yoon Lee [1,2]

[1] Department of Biomedical Sciences, Ajou University Graduate School of Medicine, Suwon, Gyeonggi 16499, Republic of Korea
[2] Institute of Medical Science, Ajou University School of Medicine, Suwon, Gyeonggi 16499, Republic of Korea

- **Supplementary Figure 1**
- **Supplementary Figure 2**
- **Supplementary Figure 3**
- **Supplementary Figure 4**
- **Supplementary Figure 5**
- **Supplementary Figure 6**

Supplementary Figure 1

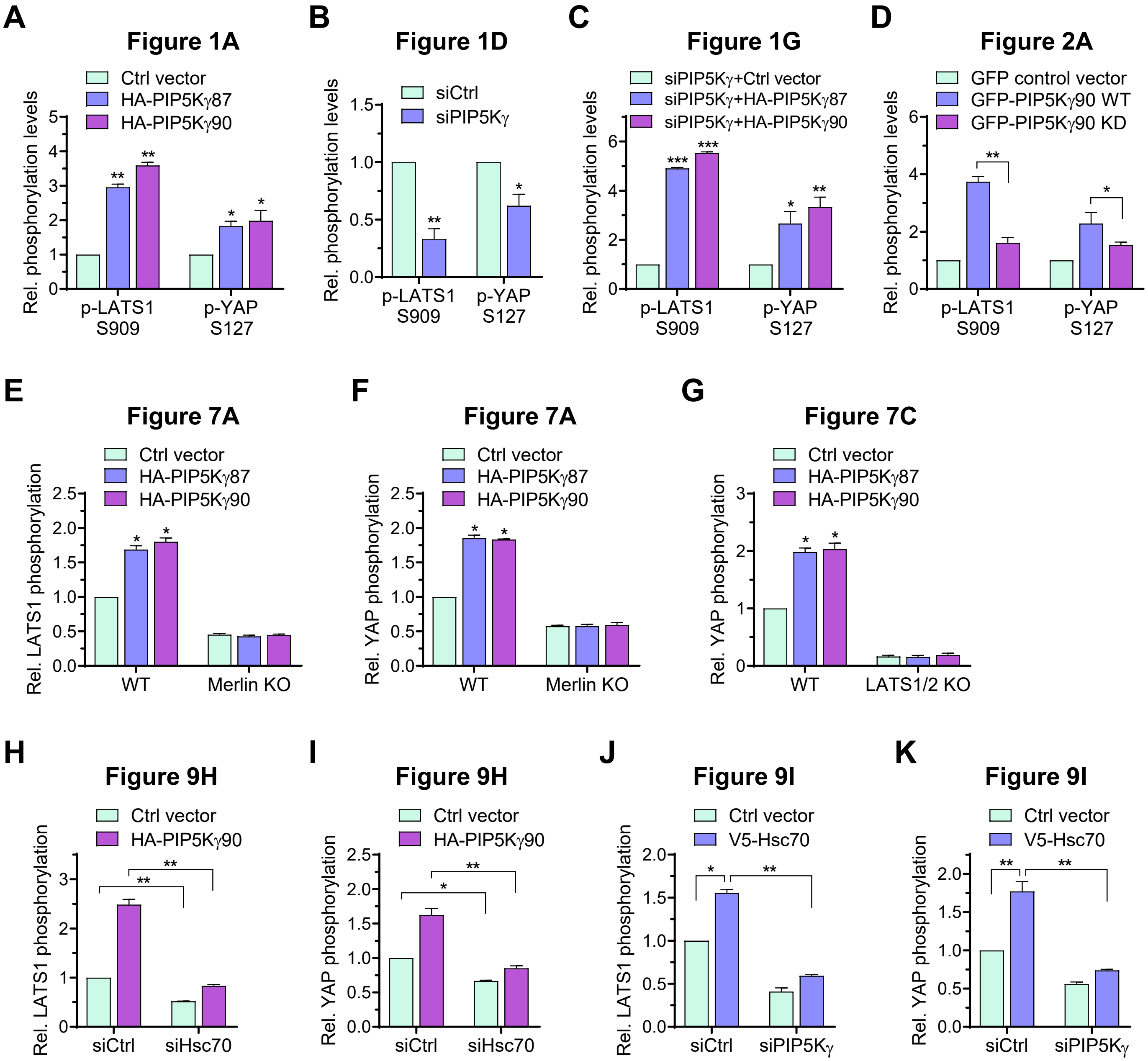

Figure S1. Quantification of phosphorylation levels of LATS1 and YAP. The phosphorylation/total ratios of LATS1 and YAP in the indicated western blot data in Figures 1, 2, 7, and 9 were quantified relative to those in corresponding control conditions ($n = 3$ each). Values in the graphs represent the mean ± S.E.M. *$p < 0.05$, **$p < 0.01$, ***$p < 0.001$.

Supplementary Figure 2

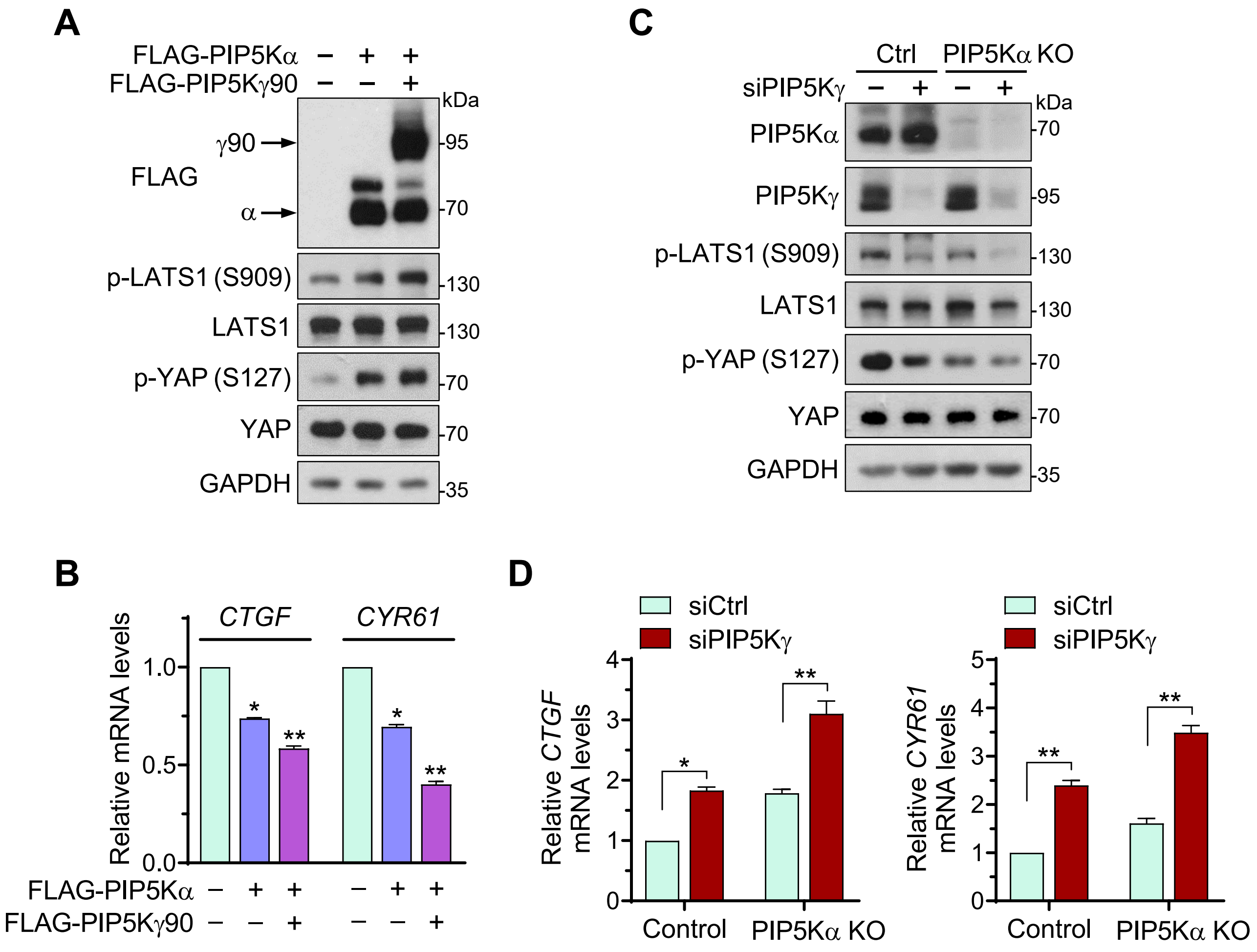

Figure S2. Comparison of the effects of PIP5Kα and PIP5Kγ on LATS1 and YAP activation. (**A**,**B**) HEK293 cells were transfected with control vector, FLAG-PIP5Kα, and/or FLAG-PIP5Kγ90. (**C**,**D**) Control and PIP5Kα KO HEK293 cells were treated with control siRNA or PIP5Kγ siRNA. (**A**,**C**) Resulting cell lysates were immunoblotted with the indicated antibodies. (**A**) Arrows indicate expressed FLAG-PIP5Kα and FLAG-PIP5Kγ90 proteins. (**B**,**D**) *CTGF* and *CYR61* mRNA levels were analyzed by qRT-PCR ($n = 3$) and relatively quantified based on those in control conditions. Values in the graphs represent the mean ± S.E.M. $^{*}p < 0.05$, $^{**}p < 0.01$.

Supplementary Figure 3

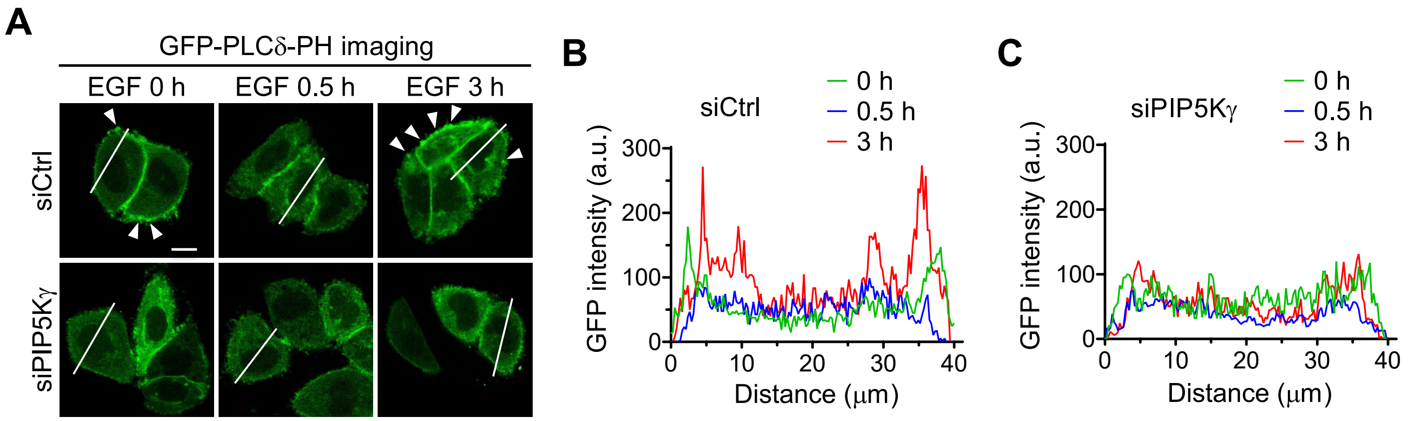

Figure S3. Quantification of GFP-PLCδ-PH images. (**A**) GFP fluorescence images in the Figure 3B were analyzed for GFP intensity profiles along the indicated lines. GFP fluorescent intensities in siCtrl (**B**) and siPIP5Kγ (**C**) conditions were measured using Zeiss ZEN imaging software and profile graphs were prepared using GraphPad Prism 8 software.

Supplementary Figure 4

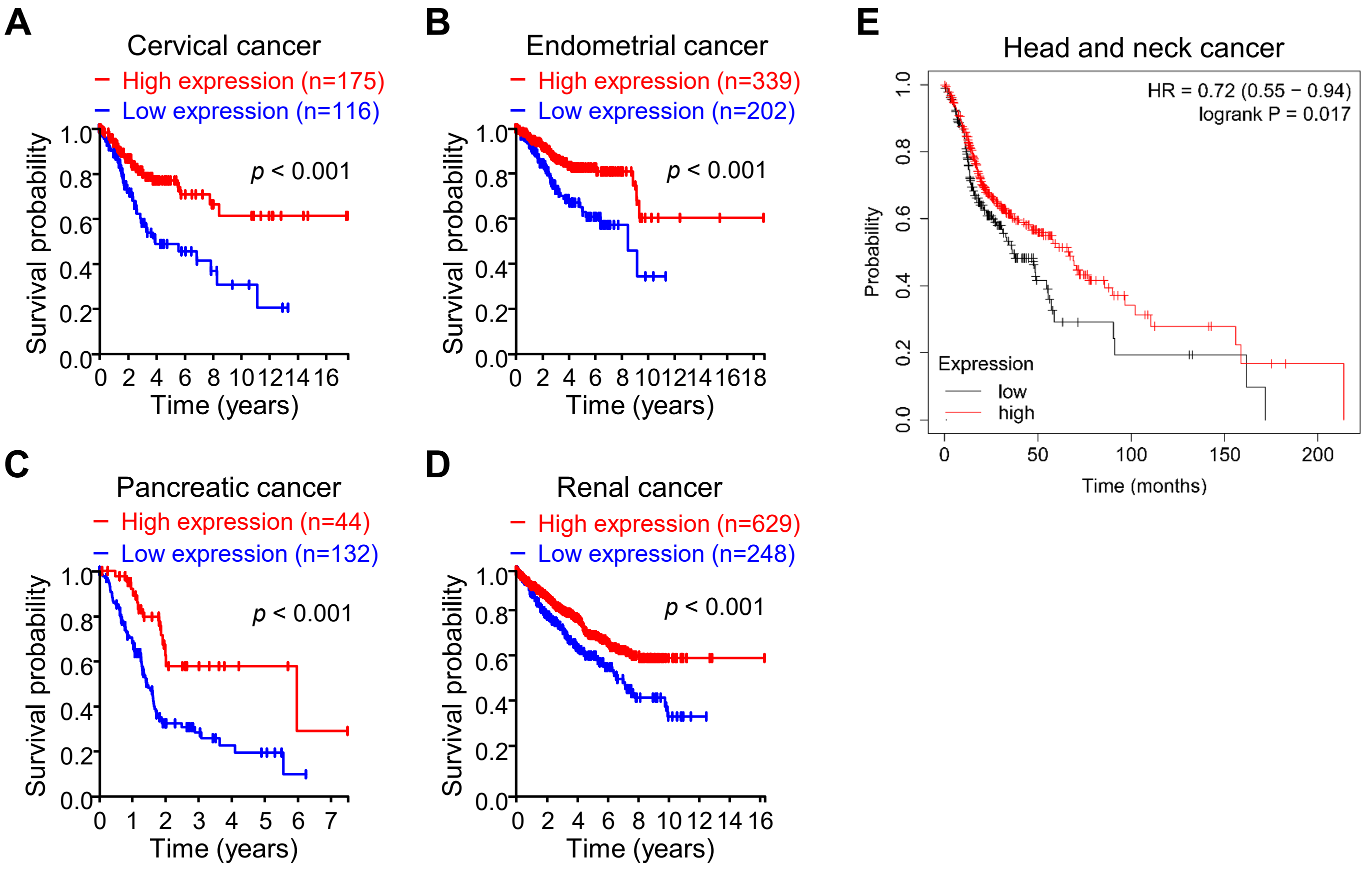

Figure S4. Kaplan-Meier survival curves of cancers associated with PIP5Kγ expression. Comparative analysis between PIP5Kγ mRNA expression levels and patient survival probability in cervical cancer (**A**), endometrial cancer (**B**), pancreatic cancer (**C**), and renal cancer (**D**) having low and high PIP5Kγ expression. Note that high PIP5Kγ expression has significant ($p < 0.001$) association with patient survival. (**A**–**D**) The graphs were slightly modified from the originals, available at: https://www.proteinatlas.org/ENSG00000186111-PIP5K1C. (**E**) Correlation between PIP5Kγ mRNA expression and overall survival probability in head and neck squamous cell carcinoma patients. The graph was available at: https://kmplot.com/analysis/. HR: hazard ratio.

Supplementary Figure 5

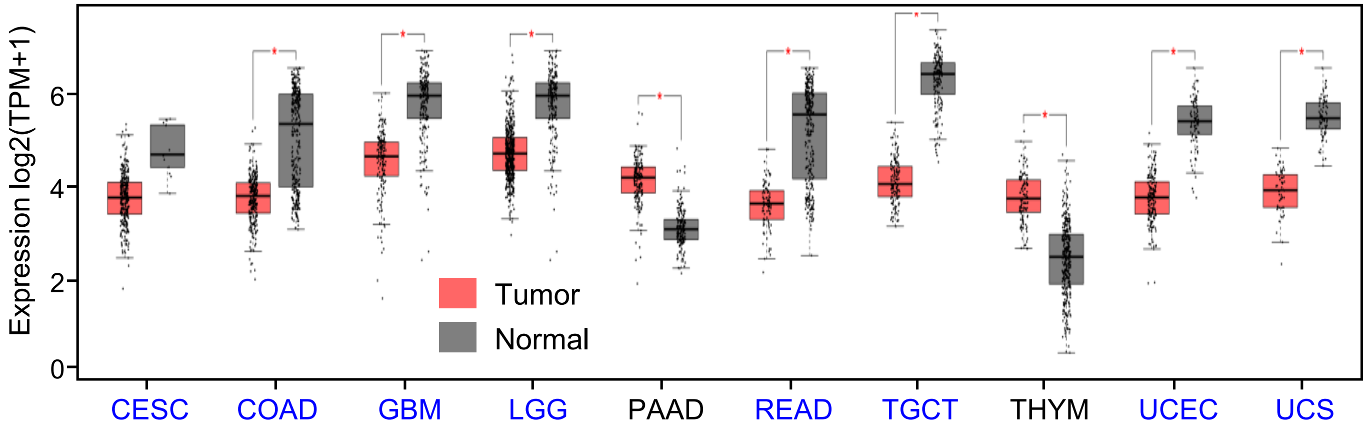

Figure S5. Gene expression profile of PIP5Kγ in various tumor samples and paired normal tissues. PIP5Kγ expression is represented as median transcripts per million (TPM) values. Blue text indicates relatively low PIP5Kγ expression in tumors, as compared with that in normal tissues. CESC, cervical squamous cell carcinoma and endocervical adenocarcinoma; COAD, colon adenocarcinoma; GBM, glioblastoma multiforme; LGG, brain lower grade glioma; PAAD, pancreatic adenocarcinoma; READ, rectum adenocarcinoma; TGCT, testicular germ cell tumors; THYM, thymoma; UCEC, uterine corpus endometrial carcinoma; UCS, uterine carcinosarcoma. The graph was slightly modified from the original, available at http://gepia2.cancer-pku.cn/.

Supplementary Figure 6

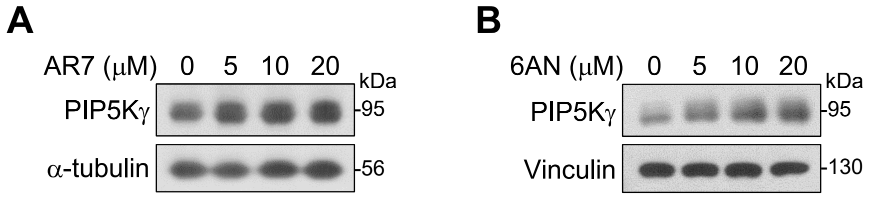

Figure S6. Effects of AR7 and 6AN treatments on PIP5Kγ protein levels. HEK293 cells were treated with different concentrations of AR7 for 12 h (**A**) or 6AN for 24 h (**B**). Resulting cell lysates were analyzed for PIP5Kγ protein levels by WB. α-Tubulin or vinculin immunoblotting was included as a loading control.
